# Endocrine regulation of *MFS*2 by *branchless* controls phosphate excretion and stone formation in *Drosophila* renal tubules

**DOI:** 10.1038/s41598-019-45269-x

**Published:** 2019-06-19

**Authors:** Emily Rose, Daniela Lee, Emily Xiao, Wenzhen Zhao, Mark Wee, Jonathan Cohen, Clemens Bergwitz

**Affiliations:** 1 Section Endocrinology, Yale School of Medicine, New Haven, CT USA; 2 Endocrine Unit, Massachusetts General Hospital, Boston, MA USA

**Keywords:** Evolutionary biology, Multihormonal system disorders

## Abstract

How inorganic phosphate (Pi) homeostasis is regulated in *Drosophila* is currently unknown. We here identify *MFS*2 as a key Pi transporter in fly renal (Malpighian) tubules. Consistent with its role in Pi excretion, we found that dietary Pi induces *MFS2* expression. This results in the formation of Malpighian calcium-Pi stones, while RNAi-mediated knockdown of *MFS2* increases blood (hemolymph) Pi and decreases formation of Malpighian tubule stones in flies cultured on high Pi medium. Conversely, microinjection of adults with the phosphaturic human hormone *fibroblast growth factor 23* (*FGF23*) induces tubule expression of *MFS2* and decreases blood Pi. This action of *FGF23* is blocked by genetic ablation of *MFS2*. Furthermore, genetic overexpression of the fly *FGF branchless (bnl)* in the tubules induces expression of *MFS2* and increases Malpighian tubule stones suggesting that *bnl* is the endogenous phosphaturic hormone in adult flies. Finally, genetic ablation of *MFS2* increased fly life span, suggesting that Malpighian tubule stones are a key element whereby high Pi diet reduces fly longevity previously reported by us. In conclusion, *MFS2* mediates excretion of Pi in *Drosophila*, which is as in higher species under the hormonal control of *FGF-signaling*.

## Introduction

Phosphate is required for many important cellular processes and having too little phosphate (hypophosphatemia) or too much (hyperphosphatemia) can cause disease and reduce lifespan in humans^1–3^. The clinical consequences of severe acute hypophosphatemia, which for example are seen in conditions of malnutrition or tumor-induced hypophosphatemia, include hemolysis, rhabdomyolysis and, in some cases, contribute to respiratory and heart failure in the intensive care unit setting^4^. Chronic hypophosphatemia leads to skeletal muscle myopathy, rickets and osteomalacia resulting in weakness, bowing and bone pain^5^. Chronic hyperphosphatemia in turn leads to renal and vascular calcification, and changes of cell metabolism which include activation of the mitogen-activated kinases ERK1/2 and mitochondrial respiration. Hyperphosphatemia is a prominent feature of rare genetic conditions such as familial hyperphosphatemic tumoral calcinosis^6^, but most frequently encountered in patients with chronic kidney disease (CKD), which currently affects 20 Million Americans, and hyperphosphatemia and elevated blood *fibroblast growth factor 23 (FGF23)* levels are important predictors of mortality in these individuals^7–9^.

In multicellular organisms, the circulating Pi levels and total body Pi content are therefore, tightly regulated by a number of hormones. These phosphate-regulating hormones include *parathyroid hormone (PTH)*, *1*,*25-dihydroxy vitamin D (1*,*25(OH)*_*2*_*D)*, *and FGF23*^3,10,11^. *PTH*, secreted by the parathyroid glands, and *FGF23*, secreted by osteocytes and in somewhat lower levels by osteoblasts^1^, lower blood Pi. These hormones do this by causing internalization of the type II sodium-Pi co-transporters *Npt2a* and *Npt2c* in the proximal tubules of the kidneys, resulting in renal losses of Pi. *1*,*25(OH)*_*2*_*D*, synthesized in the proximal tubules of the kidneys, increases *Npt2b* and *Pit2* expression in the gut, resulting in absorption of Pi from the diet, thereby increasing blood Pi. Serum Pi feeds back to regulate these hormones in an endocrine fashion with high Pi increasing the secretion of *PTH* and *FGF23* and low Pi stimulating the synthesis of 1,25(OH)_2_D^1,11^, however, it remains unclear whether “endocrine” sensing of Pi likewise requires activation of the *ERK1/2* signal transduction pathway.

Fly electrolyte homeostasis was shown to be remarkably similar to that in higher species^12–15^. Knockdown and overexpression of genes involved in fly electrolyte homeostasis have reinforced this similarity. For example, knockdown of the *dPrestin* chloride/oxalate exchanger in the Malpighian tubule, the ancestor of human renal tubules, reduced tubule calcium oxalate crystals^12^. Tubule stones ultrastructurally resemble Randall’s plaques, which are thought to be the initial event of stone formation in human kidneys^16^. If left untreated, renal stones and nephrocalcinosis are life-threatening conditions in humans. Similarly, tubule xanthine^17^, calcium oxalate^18^ and urate^19^ stone formation caused by genetic knockdown or overexpression markedly reduce fly lifespan. Using this model organism, novel insights about electrolyte homeostasis were obtained. For example, zinc was discovered to stimulate heterogeneous nucleation and stone formation in flies, a finding which is highly translatable to human nephrolithiasis and nephrocalcinosis^17^.

We recently reported that flies respond to dietary Pi during development and adult life^20,21^. When absorption of Pi is blocked by the phosphate binder sevelamer or cellular uptake is inhibited by phosphonoformic acid (PFA), larval development is delayed. The delayed development can be rescued by the addition of extra Pi to the culture medium. In contrast, adult flies exposed to high dietary Pi die prematurely. Thus, too little or too much Pi can have negative effects in flies as in humans^22^. Furthermore, if principal cells of the Malpighian tubules are genetically ablated to induce fly CKD, blood Pi (hemolymph Pi) increases and flies die prematurely. Conversely, restriction of Pi absorption from the culture medium with sevelamer extends the life span of flies with CKD. Additionally, when dietary Pi is restricted by addition of sevelamer to the culture medium or when cellular uptake of Pi is reduced after treatment with PFA, even otherwise normal adult flies live longer^21,22^.

In light of its high translational relevance, we here further characterized fly Pi homeostasis and show that a high Pi diet stimulates formation of Malpighian tubule stones. These stones likely contribute to reduced longevity of adult flies when cultured on high Pi medium. We furthermore used fly genetics and microinjection of human *FGF23* to determine the function of the fly type I sodium-Pi co-transporter *MFS2*. Among the 29 transporters identified in our earlier study^20^ as candidate Pi transporters using Bayesian phylogenetic reconstruction, *MFS2 (NaPi-T, endocoded by FBgn0016684)* mRNA is expressed highest in the Malpighian tubule and expression in the Malpighian tubules is significantly greater compared to all other tissues measured including the midgut and hindgut (Table S2). Our findings are consistent with a key role of *MFS2* mediating Pi excretion by the Malpighian tubules to lower blood Pi. This role of *MFS2* may be under the control of *FGF-signaling* to maintain blood Pi (hemolymph Pi) homeostasis as it is in higher species.

## Results

### High dietary phosphate (Pi) is accompanied by the development of hyperphosphatemia and Malpighian tubule deposits

**Figure 1:**
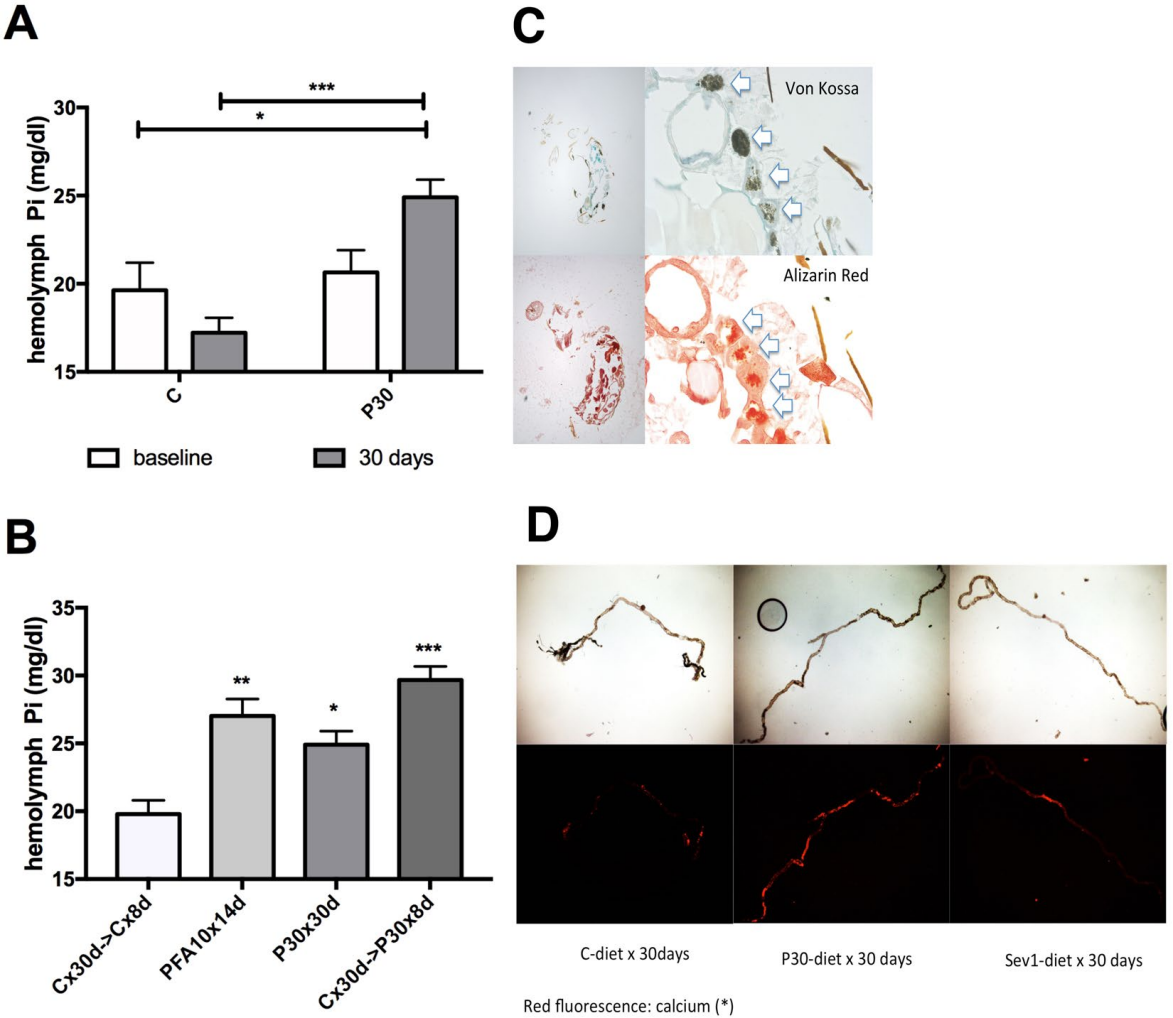
High dietary phosphate (Pi) is accompanied by the development of hyperphosphatemia and Malpighian tubule deposits. (**A**) Fly blood Pi concentration after culture of *y w* females at 25 °C on control (C) alone or medium supplemented with 30 mM sodium phosphate (P30) for five (baseline) and 30 days, (mean ± SEM, n = 4 pooled collections of 15 flies, ***p = 0.0002, *p = 0.0424). (**B**) Fly blood Pi concentration after culture of *y w* females at 25 °C on control medium (C) for 30 days, followed by C or P30 medium for 8 days, or culture on C medium supplemented with the Pi transport inhibitor phosphonoformic acid 5 mM (PFA) for 14 days (mean ± SEM, n = 4–8 pooled collections of 15 flies, ***p = 0.0001, **p = 0.0023, *p = 0.0110). (**C**) Paraffin embedded sagittal sections of adult flies after culture for 30 days on P30-medium stained for Pi with von Kossa/methylene green (black stain in upper panels) or for calcium with Alizarin Red (red stain in lower panels) (4X (left) and 40X (right)). (**D**) Medium supplemented with 1% sevelamer (Sev1) reduces these calcium Pi deposits, when compared to culture on C and P30 medium. Alizarin Red stain of native preparations of anterior Malpighian tubules (representative of 4–8 tubule pairs).

To begin to understand why flies die when exposed to high dietary Pi^21,22^ we first determined hemolymph (blood) Pi in young and in aged flies on standard medium (C), medium supplemented with 30 mM sodium phosphate (pH 6.0) (P30) or medium supplemented with 5 mM phosphonoformic acid (pH 6.0) (PFA), an antiviral drug that also blocks Pi transporters, and which extends fly life span^21^. We observed that young flies maintain normal hemolymph Pi levels on P30 medium at young age while exposure of adults to P30 for 30 days raised blood Pi (Fig. 1A). Even exposure of 30-day-old flies for 8 days to P30 raised blood Pi (Fig. 1B). These findings suggest that homeostatic mechanisms are in place that keep blood Pi stable in young flies. These homeostatic mechanisms deteriorate over their lifespan and lead to hyperphosphatemia.

We next postulated that hyperphosphatemia may reduce life span by causing tissue mineralization, as previously described^23^. To look for tissue mineralization we used paraffin sections obtained from adult flies and the von Kossa Pi stain and the alizarin red calcium stain (Fig. 1C). We observed physiological formation Ca-Pi deposits in the Malpighian tubules that are the ancestors of renal tubules in higher species^24^. The alizarin red stain can also be detected by epifluorescent technique in native tubule preparations (Fig. 1D). Using these staining modalities, we observed that Ca-Pi deposits increased after culture of flies on P30 medium. In contrast, they decreased when flies were cultured on medium containing 1% sevelamer, a polycation that modulates serum phosphate by binding to Pi and reducing its absorption from the diet in humans with CKD^25^.

### Tubule deposits co-localize with stellate cells and consist of microspheres that contain calcium and phosphate

**Figure 2:**
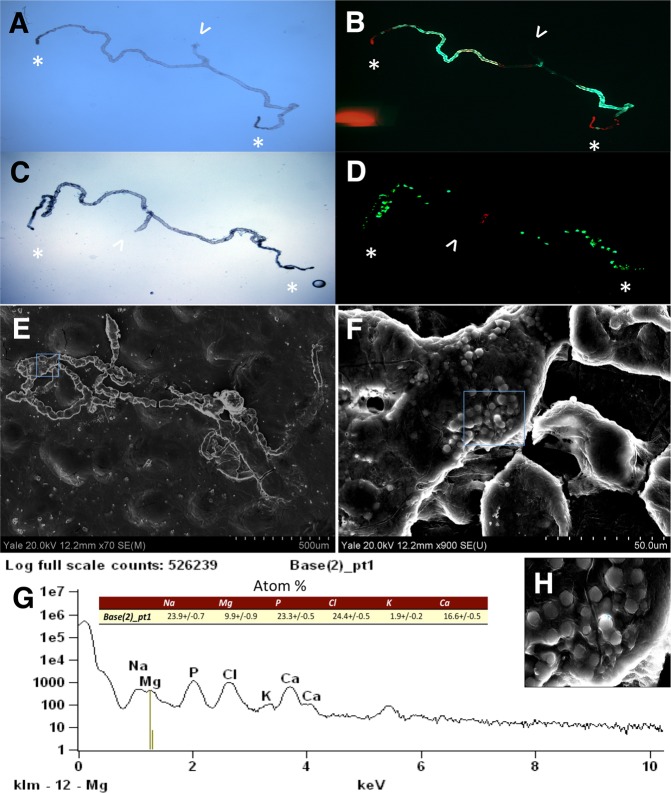
Tubule deposits co-localize with stellate cells and consist of microspheres that contain calcium and phosphate. (**A**,**C**) Bright field and (**B**,**D**) epi-fluorescent micrographs (right) of native preparations of anterior Malpighian tubules from flies expressing GFP in principal (**A**,**B**) or stellate cells (**C**,**D**). Alizarin Red is used to stain for calcium (arrow points to collecting duct, *marks calcium in distal tubule). Scanning electron microscopic (SEM) images of anterior Malpighian tubules from a fly cultured on P30 for 30 days (70X, **E**; 900X, **F**), followed by energy-dispersive X-ray-spectroscopy (EDX) microanalysis in **G** of an individual microsphere shown in the inset (2700X, **H**).

Ca-Pi deposits co-localize with the distal, stellate cell-rich segment of the tubules (Fig. 2B,C) but will also fill the proximal, principal cell-rich segment of the tubules when flies are cultured on P30 medium (Fig. 2D,E). Scanning electron microscopic analysis showed microspheres, which are typical for Ca-Pi (apatite) (Fig. 2F,G), and similar to those seen in human Randall’s plaques as previously reported for Ca-oxalate tubule stones^16^. Mineral composition was analyzed by energy-dispersive X-ray-spectroscopy (EDX) microanalysis and confirmed presence of calcium and phosphorus in individual microspheres (Fig. 2H,I).

### Tubule calcium-phosphate deposits form in anterior tubules, increase with age and at high culture temperature

**Figure 3:**
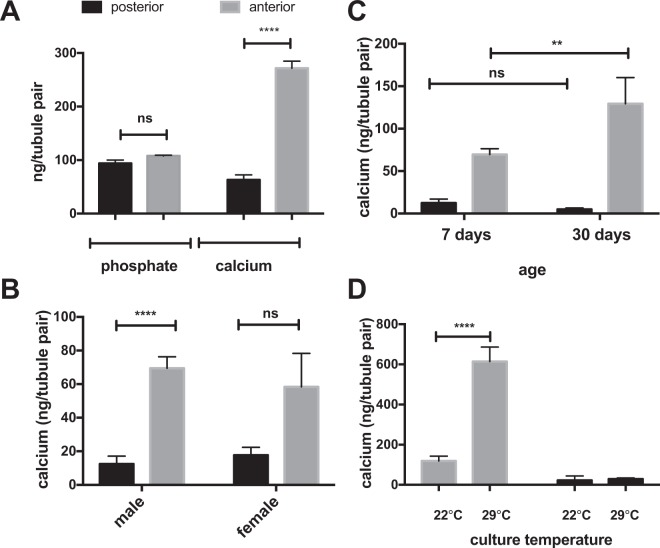
Tubule calcium-phosphate deposits form in anterior tubules, increase with age and at high culture temperature. Anterior and posterior Malpighian tubule pairs were dissected from adult males (**A**,**C**,**D**), or young adult males and females (**B**) that had been cultured for 7 or 30 days at 22 °C or 29 °C as indicated on the x-axis. The Pi and calcium (**A**) or calcium content (**B**–**D**) in each tubule pair were measured following tubule dissection and complete lysis of the tissue (mean ± SEM, n = 4–8 tubule pairs, ****p < 0.0001, **p = 0.002).

To quantify the Ca-Pi deposits we initially attempted to measure Pi content of Malpighian tubule preps directly, but found, that abundant intracellular Pi released during tubule cell lysis adds to that measured in Ca-Pi deposits which considerably interferes with this assay (Fig. 3A). Conversely, tubule calcium, which is known to be several orders of magnitude lower inside tubule cells, corresponded closely to the extent of extracellular tubule Ca-Pi deposits observed microscopically. Consistent with findings reported by others^26^ we furthermore observed that anterior tubules have 10-fold more deposits compared to posterior tubules (Fig. 3B). While there is no difference between males and females (Fig. 3B), anterior tubule calcium increases with age (Fig. 3C), and with culture temperature from 22 °C to 29 °C (Fig. 3D). At higher culture temperatures, flies consume more water and become dehydrated more easily^27^. Increasing age and dehydration in the setting of higher ambient temperatures are both known kidney stone risk factors in humans^28,29^.

### *MFS2* is highly expressed in the Malpighian tubules of y w flies, induced by dietary Pi and ablation of *MFS2* increases fly blood Pi and prolongs life span

**Figure 4:**
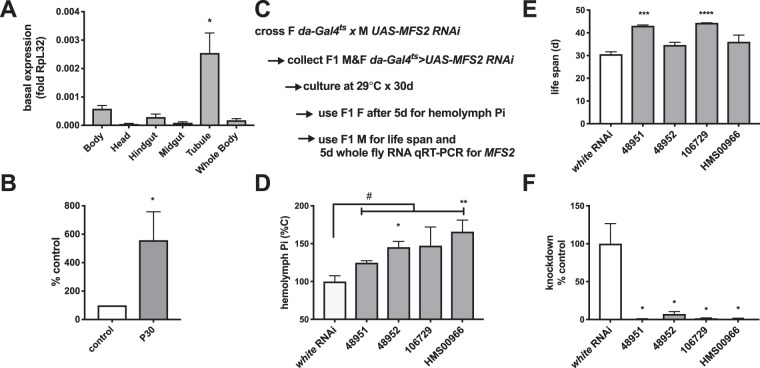
*MFS2* is highly expressed in the Malpighian tubules of *y w* flies, induced by dietary Pi and ablation of *MFS2* increases fly blood Pi and prolongs life span. (**A**) qRT-PCR for *MFS2* mRNA in fly tissues after culture of *y w* adults on control medium (mean ± SEM, n = 7–40, *p = 0.01 vs. Whole Body). (**B**) qRT-PCR for *MFS2* mRNA in Malpighian tubules dissected from *y w* adults after culture on control and P30 medium for 5 days (mean ± SEM, n = 5, *p = 0.04 vs. control). (**C**) when *MFS2* is ablated universally using *da-Gal4*^*ts*^ driving expression of four different transgenic RNAis targeting *MFS2* (TRIP or VDRC ID indicated on the x-axis) blood Pi levels are increased after culture at 29 °C for 5 days (mean ± SEM, n = 4–20 pooled collections of 15 female flies, ^#^p = 0.002, **p = 0.002, *p = 0.04 vs. *white-RNAi*). (**D**) Male siblings were used to determine effect of *MFS2* ablation on life span (mean ± SEM, n = 51–381 flies, **p = 0.002, *p = 0.03 vs. *white-RNAi)* (**E**) and to confirm *MFS2* knockdown (**F**) (mean ± SEM, n = 4 flies, *p = 0.03 vs. *white-RNAi)*.

Interestingly, PFA raises blood Pi (Fig. 1B), suggesting that this drug blocks excretion of Pi in the Malpighian tubules (Fig. S1A). We next searched FlyAtlas^30^ for candidate PFA-sensitive Pi transporters. Among the 29 transporters identified in our earlier study as candidate Pi transporters using Bayesian phylogenetic reconstruction^20^, *MFS2 (NaPi-T/FBgn0016684)* mRNA is expressed highest in the Malpighian tubules. The expression of *MFS2* in the Malpighian tubules is over 500 times that in the midgut, and over 1000 times that in the hindgut. *MFS2* expression in the Malpighian tubules is similarly significantly greater compared to all other tissues measured (Fig. S1B and Table S2). High expression of *MFS2* in the tubule was confirmed using qRT-PCR (Fig. 4A) and found to be stimulated by dietary Pi (Fig. 4B), consistent with an excretory function for Pi. Furthermore, RNAi-mediated ablation of *MFS2* in all fly tissues using the temperature-inducible gene expression system^31^ and *daughterless (da) Gal4*^*ts*^ as driver increased blood Pi (Fig. 4D) when flies were cultured at the permissible temperature for 5 days. Based on these findings we postulated that *MFS2* may negatively regulate hemolymph Pi by increasing excretion of Pi in the Malpighian tubules. Similar to chemical inhibition of Pi excretion with PFA as previously reported by us^21^, ablation of *MFS2* increased fly life span in two of the four *MFS2-RNAi* lines (Fig. 4E).

### Microinjection of human *FGF23* induces *MFS2* expression and reduces fly blood Pi, which is blocked by RNAi mediated ablation of *MFS2*

Many signaling pathways identified in *Drosophila* are conserved in higher species and in humans^32^. A homology search using the DIOPT tool^33^ revealed one FGF-like peptide (*branchless bnl)*, two FGF-receptors (*breathless*, *btl and heartless*, *htl*), and one klotho (KL)-like protein (*CG9701*) encoded in the *Drosophila* genome, while orthologs of parathyroid hormone (PTH) and PTH-related peptide (PTHRP), that also regulate Pi excretion in higher species^3^, are missing in flies^20^ (Table S3). Two less well studied FGF-8-like peptides were not identified in the DOIPT search likely due to lower homology to *FGF23* than *bnl* (Fig. S3).

**Figure 5:**
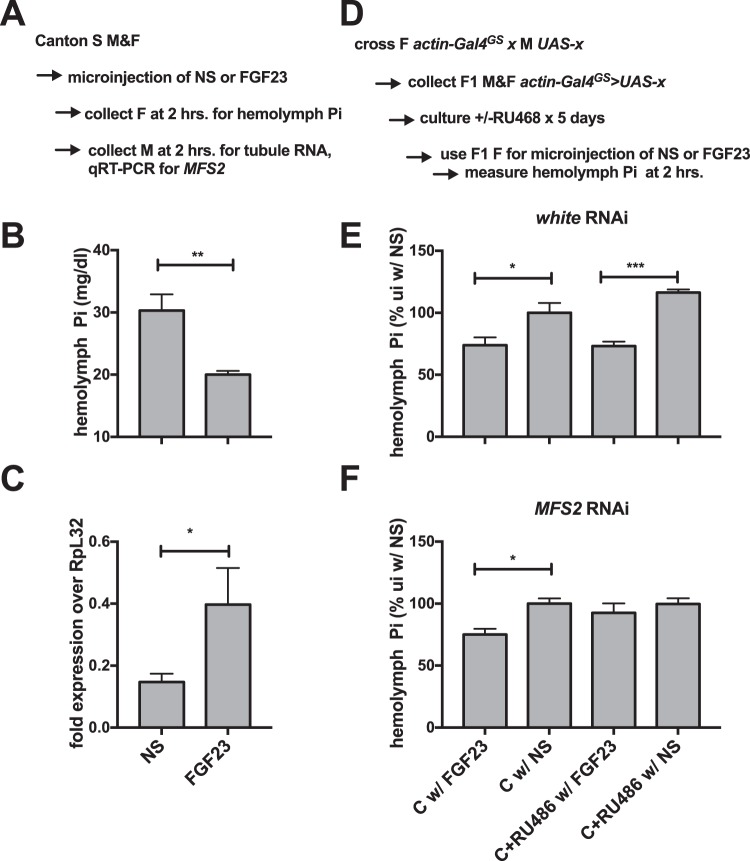
Microinjection of human *FGF23* induces *MFS2* expression and reduces fly blood Pi, which is blocked by RNAi mediated ablation of *MFS2*. (**A**) Two hours after microinjection (NS, 100 nl 0.9% sodium chloride; *FGF23*, 100 nl *FGF23* 20 ng/ml) *CS* female adults were collected for hemolymph Pi (**B**) while CS males were used for preparation of mRNA of Malpighian tubules to determine expression of *MFS2* by qRT-PCR (**C**). (**D**) Flies with the genotypes *actinGal4*^*GS*^ > *UAS-w RNAi (TRIP HMS00017*, (**E**) or *actinGal4*^*GS*^ > *UAS-MFS2 RNAi (TRIP HMS00966*, (**F**) were cultured on control medium w/ or w/o 150 ng/ml RU486 to induce global RNAi-mediated knockdown for 5 days followed by hemolymph collection from females two hours after microinjection with *FGF23* or NS. (mean ± SEM, n = 4–5, ***p = 0.0001, **p = 0.0023, *p = 0.0110).

Since fly *bnl* peptide is not readily available and many compounds and hormones that are used in humans are also active in flies^34,35^, we decided to test the hypothesis that human *FGF23* is active in flies as a phosphaturic hormone. 2 hours following microinjection of adult flies with human recombinant *FGF23* we collected hemolymph and dissected Malpighian tubules for total RNA extraction. When compared to vehicle injected flies, human recombinant *FGF23* decreased hemolymph Pi (Fig. 5B), possibly as a result of increased mRNA expression of *MFS2* (Fig. 5C) and increased excretion of Pi by the tubules. This effect of *FGF23* was blocked by RNAi-mediated ablation of *MFS2* using the universal gene-switch driver *actin-Gal*^*GS*^, but not in uninduced flies or flies in which the *white* gene had been ablated (Fig. 5E,F).

### Ablation of *MFS2* and overexpression of *bnl* in principal cells decreases or increases Malpighian tubule deposits, respectively

**Figure 6:**
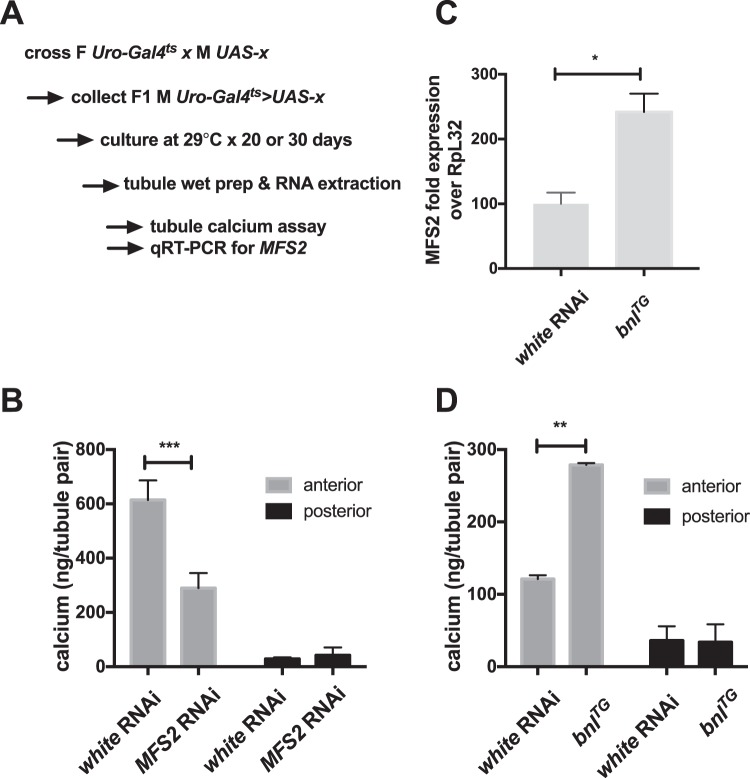
Ablation of *MFS2* and overexpression of *bnl* in principal cells decreases or increases Malpighian tubule deposits, respectively. (**A**) Flies expressing the tubule driver *Uro-Gal4*^*ts*^ and RNAi targeting *white (w)* as control and *MFS2* (**B**), or a transgene (TG) for *bnl* (**C**,**D**) were generated and cultured on control medium at 29 °C to induce gene expression for 20–30 days as shown in the scheme, followed by dissection and complete lysis of anterior and posterior Malpighian tubule pairs for calcium assay (**C**), or RNA preparation followed by qRT-PCR for *MFS2* (D) (mean ± SEM, n = 4–5, **p = 0.002, ***p = 0.0002 vs. *white* RNAi).

We finally used the inducible tubule-specific drivers *Uro-Gal4*^*ts*^ and *Gal4c724*^*ts*^ for ablation of *MFS2* in principle and stellate cells, respectively. Adult flies cultured at the permissive temperature for 30 days showed no microscopic difference in tubule deposits when targeting *MFS2* in stellate cells (Fig. S2B). However, ablation of this transporter in principle cells reduced tubule Ca-Pi deposits microscopically (Fig. S2C), which was confirmed using the tubule calcium assay (Fig. 5B). Furthermore, overexpression of a *bnl* transgene increased expression of *MFS2* in tubule RNA obtained from flies cultured at the permissible temperature for 5 days (Fig. 6C). As a consequence, tubule calcium content (Fig. 6D) and mineralization observed microscopically in native tubules was increased (Fig. S2D).

**Figure 7:**
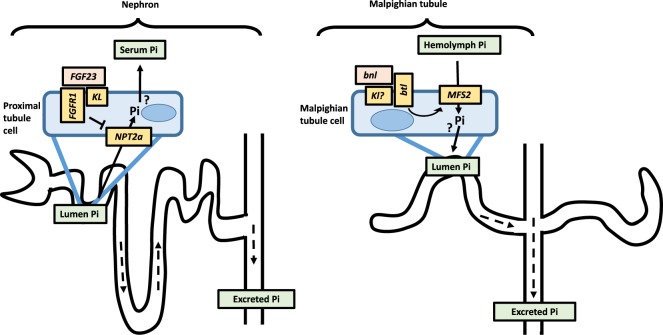
Summary. Based on expression in the tubule and stimulation by dietary Pi, *MFS2* may function as excretory Pi-transporter in Malphighian tubules (right panel), a difference that may reflect the unique anatomy of the fly renal system which lacks connection to a glomerulus seen in higher species (left panel). We further show here that *bnl* stimulates Pi excretion by *MFS2* which increases tubule stone formation, while ablation of *MFS2* increases hemolymph Pi and blocks formation of tubule stones. Figure design after^13^.

These results are consistent with a key role of *MFS2* mediating Pi excretion by the Malpighian tubules. *MFS2* lowers blood Pi in adult flies and may be, as in higher species, under the control of *FGF-signaling* to maintain hemolymph Pi homeostasis (Fig. 7).

## Discussion

We previously reported that raising dietary phosphate (Pi) reduces life span of *Drosophila* by 30%^21,22^, and show here that this life span reduction is accompanied by elevated fly blood (hemolymph) Pi and an increase of renal (Malpighian tubule) stones. Since treatment with PFA and ablation of *MFS2* improved life span despite worsening the hyperphosphatemia, our results suggest that the primary cause of death of flies when cultured on high Pi medium is the formation of tubule stones. Although the exact mechanism is unknown, tubule stone formation may reduce Malpighian tubule function and induce fly chronic kidney disease (CKD), as has previously been suggested for flies developing xanthine, Ca-oxalate or urate stones, who likewise die prematurely^17–19^. Furthermore, our findings are consistent with GWAS data which identified the proximal tubular sodium Pi co-transporter *NPT2a* as a determinant of renal function in humans^36,37^. Additionally, our work corroborates data in mice and humans who develop potentially life-threatening renal calcifications, and sometimes chronic kidney disease, due to loss-of-function mutations in *NPT2a* and the related isoform *NPT2c*^38–43^. Therefore, hyperphosphaturia in flies and higher species reduces longevity by causing kidney stones.

Since we treated the entire organism with PFA and universally ablated *MFS2* it is also possible that we reduced Pi toxicity in other tissues in addition to the Malpighian tubules. We also may have caused “compensated hyperphosphatemia” as has been described in human early stage CKD 2 and 3. This in turn causes maladaptive endocrine changes such as elevation of *fibroblast growth factor 23 (FGF23)*, suppression of 1-alpha hydroxylation in the kidney and development of secondary hyperparathyroidism in humans and reduce longevity^44^. Consistent with this conclusion, we showed previously that genetic impairment of Malpighian tubule function to induce fly chronic kidney disease (CKD) reduces adult life span^21^. Longevity was improved by supplementation of culture medium with 1% sevelamer, which normalizes hemolymph Pi in these CKD flies.

Our observation that high Pi diet increases naturally occurring Ca-Pi deposits in anterior tubules extends earlier studies of anterior-specific expression of the calcium channel *Best2* and the peroxisomal Ca^2+^/Mn^2+^ ATPase isoform C *SPoCk-C*^45^. However, the observation that *MFS2* has excretory function for Pi in the Malpighian tubules was at first sight unexpected, since Pi transporters in higher species are re-absorptive (Fig. 7, left panel). Knockout of *Npt2a* for example leads to hyperphosphaturia in mice^46^ while our principal cell-specific ablation of *MFS2* led to hypophosphaturia. However, this difference may reflect the unique anatomy of the fly renal system which lacks connection to a glomerulus (Fig. 7, left panel) and consequently depends on secretory transporters (Fig. 7, right panel) to maintain phosphate homeostasis^13,24^. That ablation of *MFS2* blocks formation of Ca-Pi deposits is furthermore similar to the effect of ablation of the fly oxalate, xanthine or urate transporters, that blocks stone formation. It may be of interest to further investigate where Pi and Ca excretion by the Malpighian tubules are co-regulated, and since Ca-Pi is thought to form the initial nidus of many stone disorders, whether ablation of *MFS2* reduces formation of oxalate, xanthine or urate stone formation.

*MFS2* is related to yeast *Pho84* and human *NPT1* and like *NPT1* uses the transmembrane sodium gradient to transport Pi against its electrochemical gradient into cells. It therefore would be predicted to localize to excretory vesicles of principal cells. It is also possible that Pi is transported transcellularly like in proximal tubular cells of higher species, imported by *MFS2* at the basal membrane of principal cells and then excreted into the fly urine via apical Pi exporter(s), for example the fly ortholog of *Xenotropic retroviral receptor (Xpr1*, *CG10483)*.

Malpighian tubule stones may in the absence of a skeleton serve as Ca-Pi stores in flies in addition to their excretory function, as has been suggested^26^. To permit re-absorption of Pi in periods of limited dietary intake, apical transporters might be needed, for example one of the 29 *MFS* transporters previously reported by us^20^, or the single type III Pi transporter *dPit (NaPi-III)*, which is subject of future investigation.

Since *MFS2* mRNA is detected at low levels in tissues other than the Malpighian tubules (Table S2) it is finally possible that *MFS2* contributes to dietary absorption of Pi or as sensor for Pi in cells secreting *bnl* (see below). However, our finding that principal cell-specific ablation of *MFS2* reduces stone formation argues strongly suggests that *MFS2*’s role in Pi balance is dependent on its presence in the Malpighian tubules rather than in other tissues.

Many signal transduction pathways have been discovered in *Drosophila* and are found to be highly conserved to higher species and in humans^32^. Since flies lack orthologs for *PTH* and *PTHRP*, we postulated that FGF-signaling may regulate blood Pi in flies. This hypothesis is supported by our finding that injection of human *FGF23* increases tubule *MFS2* expression and lowers blood Pi. This effect is blocked by genetic ablation of *MFS2*. Furthermore, transgenic overexpression of *bnl* in principal cells increases expression of *MFS2* and Malpighian tubule deposits. Therefore, *bnl* may double as phosphatonin in adult flies (Fig. 7, right panel), and it will be of interest down the road to study *MFS2* gene expression in response to recombinant fly *bnl* protein or their response to dietary Pi after ablation of *bnl* to prove that the Pi effect on *MFS2* is dependent on *bnl*.

Flies lack a skeleton, which is the source of the mammalian phosphatonin *FGF23*. Therefore, *bnl* may be secreted by the gut and/or the Malpighian tubules which both highly express *bnl* according to FlyAtlas^30^. Since the fly brain secretes *Drosophila insulin-like peptides* and other neuropeptides^47^ it may also be of interest to look for *bnl*-secreting phosphoregulatory neurons.

Among other candidate hormones which may have a role in fly mineral homeostasis are *pyramus (pyr)* and/or *thisbe (ths)* which are less well studied FGF-8-like peptides with lower homology to *FGF23* than *bnl* (Fig. S3), that bind to *heartless (htl)* and regulate the development of somatic and visceral muscle in the mesoderm, cardioblasts and glial cells^48^. In addition, vitamin D synthesizing enzymes are found in the fly genome. It is also possible that the steroid hormone ecdysone, which regulates the transition from embryo into larva and then into pupa^49^, activates orthologs of mammalian vitamin D receptor in adult flies. Finally, our DIOPT search indentified a fly ortholog of mammalian alpha klotho (Table S3). Alpha klotho is essential for FGF23-signaling in higher species (Fig. 7, left panel)^1^. It may therefore be of interest to test whether *pyr*, *ths*, *fly klotho* and/or *ecdysone* have a role in adult fly Pi homeostasis in addition to *bnl* in future experiments.

In summary, our findings suggest that Malpighian tubule stones, not hyperphosphatemia, reduce fly longevity caused by high Pi diet. They are furthermore consistent with a key role of *MFS2* for mediating Pi excretion by the Malpighian tubules to lower blood Pi, which may be as in higher species under the control of *FGF-signaling* (Fig. 7).

Our findings have several translational aspects: As in flies hyperphosphaturia can lead to stone formation under certain conditions in humans^50^. Therefore, *Drosophila* drugs screens could identify novel compounds that modify Malpighian tubule stone formation which also have activity in human stone disease. Our findings furthermore raise the intriguing possibility that excretory Pi transporters exist in the kidneys of humans. These transporters may be re-activated in states of disease and could be of interest as drug targets under certain conditions. Finally, we recently reported serval genes whose ablation in adult flies changes hemolymph Pi levels and longevity^21^. If these genes modify *bnl* and *MFS2* gene expression, response to microinjection of human *FGF23* and tubule stone formation in flies, then their human orthologs could be used to identify novel drugs that also reduce morbidity and mortality in the human population with chronic kidney disease (CKD), which currently affects 20 Million Americans, and for whom dietary Pi is an important predictor of mortality^7–9^.

## Methods

### Fly culture, genotypes and crosses

Standard fly culture was performed at 22 °C on (per vial) 0.1 g agar, 1.2 ml molasses, 0.9 g corn meal, 0.1 g dry yeast in 15 ml water to which 4 ml/l propionic acid and 250 mg/l tegosept (Spectrum M1187) are added as mold inhibitors.

This medium was supplemented with 30 mM sodium phosphate (pH6.0), 1% sevelamer (a gift by Sanofi, Inc., Framingham, MA) or 10 mM phosphonoformic acid (Sigma P6801). The soluble fraction of Pi in SM is about 3.7 mg/dl (1.2 mM), and supplementation with 1% sevelamer reduced it to 0.16 mg/dl (0.05 mM), while 30 mM sodium phosphate (pH 6.0) raised the soluble inorganic Pi to 110 mg/dl (35 mM)^21^. Previous tests with equimolar concentrations of sodium sulfate and dye-feeding assays^51^ showed absence of non-specific of effects due to differences in osmolarity and preferential consumption of supplemented foods^21^.

For mifepristone-inducible gene regulation^52^ we used *P{Act5C(-FRT)GAL4*.*Switch*.*PR}*^3^ (*actin-Gal4*^*GS*^*)* and a final concentration of 150 ug/ml mifepristone (R486)(VWR #TCM1732-5G). The temperature-regulated *Gal80*/*Gal4* driver stocks^31^ used here were: *w-*, *hs-hid(y)/w-;tub-Gal80t*^*s20*^*;da-Gal4 (da-Gal4*^*ts*^*)*, *w-;Uro-Gal4/flCyO;tub-Gal80*^*ts7*^ *(Uro-Gal4*^*ts*^*) and Gal4*^*c724*^*(II)/CyO;tubGal80*^*ts7*^ *(Gal4*^*c724*,*ts*^*)*. *The principal cell driver w-;Uro-Gal4/CyO* and the stellate cell driver *w-;Gal4* ^*c724*^*(II)* to generate these stocks were kindly provided by Julian Dow, University of Glasgow, UK. Transgenic flies carrying RNAi constructs were obtained from the Transgenic RNAi Project (TRiP)^53^ and Vienna Drosophila RNAi Center (VDRC)^54^. The transgenic overexpression constructs are *GFP*^*TG*^: w*;P{UAS-2xEGFP}AH2 (Bloomington ID #6874); bnl^*TG*^*: w*, *UAS-bnl;Sco/CyO*^55^. *yellow white* (*y w*) and *Canton S* flies were used as wildtype stocks. Fly RNAi lines targeting *white* (TRiP #HMS00017) and *GFP* (Bloomington ID # 35785) were used to control for non-specific RNAi effects. Off-target effects were unlikely, if results were reproducible by at least two independent RNAi-lines and significant based on p < 0.05 (Student’s t-test or ANOVA with Tukey’s test for multiple comparisons) across all RNAi-lines targeting a single gene when compared to control hairpins targeting *white or GFP* and at uninduced or induced condition. The inducible driver line was validated with each replicate experiment in a parallel cross with the *GFP*^*TG*^ line. Knockdown efficiency was confirmed by qRT-PCR and some subsequent experiments were performed only using the strongest RNAi line. Genetic background effects were unlikely since phenotypes were generally absent in the uninduced state.

### Microinjection of flies

Using a micromanipulator (World Precision Instruments, Sarasota, FL) to guide a positive displacement pipette (Drummond, scale 0.1–10 ul), a Nikon dissection microscope (4–10 X magnification) and untreated glass capillaries shaped using a micropipette puller (World Precision Instruments, Sarasota, FL) we microinjected 100 nl of normal saline (NS, 0.9% NaCl) or NS containing 20 ng human *FGF23*/ml in rapid sequence into the abdomen of flies immobilized on a CO_2_ pad^56^. Using this approach, it is possible to inject 20–30 flies within 1–2 minutes, which then were transferred into a clean culture tube with standard medium with or without mifepristone (R486). Pilot experiments showed very little lethality following microinjection and flies appeared to behave normally when observed for a week following the injection. However, flies were generally analyzed two hours after the injection as described below. Human *FGF23* was provided by Susan Schiavi, Genzyme Inc. and tested on Western for integrity and able to induce hypophosphatemia in mice. *FGF23* stocks were dissolved at 0.5 mg/ml 0.01 M acetic acid and stored at −70 °C prior to dilution to working stocks in NS. Assuming a total blood volume of flies of 2 ul injection of 100 nl of 20X *FGF23* (20 ng/ml) will obtain a final concentration of 1 ng/ml, which 10-fold in excess of human or mouse concentrations of *FGF23* (~0.1 ng/ml).

### qRT-PCR, hemolymph Pi and Malpighian tubule calcium and Pi assays

After previous tests comparing males and females had ruled out gender-specific effects of dietary Pi supplementation or RNAi-knockdown^21,22^, we typically used F1 generation males for gene expression analysis and their female siblings for hemolymph collection. The animals were cultured at permissive conditions for five to thirty days as indicated.

5 bodies, 10 heads, 20 guts or 20 tubule pairs, were dissected from F1 males for total RNA preparation using Trizol reagent (Invitrogen). cDNA was synthesized using 150 ng total RNA per reaction and the Omniscript cDNA reverse transcription kit (Qiagen). The levels of mRNA for different genes were measured by using SYBR-GREEN QuantiTect (Qiagen) on a StepOnePlus real time PCR system (Applied Biosystems). The primers used are listed in Table S1. *RpL32* (*FBgn0002626*) was used for normalization, which, unlike *actin5C*, is not influenced by culture temperature of the flies^57^.

For hemolymph collection 15–20 females were anesthetized with CO_2_, heads were removed, and fly bodies were centrifuged at 5000 rpm for 3 min. at 4 °C in a 200 ul polypropylene vial with a punctured bottom, allowing for the collection of approximately 2 ul clear, cell-free hemolymph in a 500 ul polypropylene vial. 0.5 ul hemolymph was further diluted in 10 ul dH_2_O and stored at −70 °C until samples were then assayed for Pi in 100 ul ammonium molybdate phosphate assay reagent (Phospho Liqui-UV, Stanbio 0851-250) at 340 nm.

Anterior and posterior Malpighian tubule pairs were dissected in ice-cold PBS containing 1% of BSA, which prevents tubule stones from dissolving during dissection and tubules from getting stuck in pipette tips used to transfer individual pairs into a 96-well plate containing 10 ul 1 M HCl for hypotonic lysis of cells and to dissolve tubule stones. The level of calcium in each tubule pair was measured using Calcium Liqui UV (Stanbio #830) at 560 nm. We also tested the level of Pi in each tubule pair using Stanbio Phospho Liqui UV (Stanbio#0851-250) at 340 nm.

### Histological and scanning electron microscopic evaluation

Paraffin embedding of adult flies fixed in phosphate buffered saline (PBS) formalin for 30 min. was performed under vacuum to permit penetration of the medium, followed by 10 um sectioning and staining with von Kossa/methylene green (black stain in upper panels) or for calcium with Alizarin Red (red stain in lower panels) using standard protocols (4X and 40X magnification). Alizarin Red staining of dissected Malpighian tubules was performed in PBS containing 0.1% Triton X100 and 20 mg/ml Alizarin Red dye, followed by bright field and epi-fluorescent micrographs of life Malpighian tubules from flies expressing GFP in principal or stellate cells at 360/670 nm and 360/488 nm (excitation/emission wavelength), respectively. For scanning electron microscopy Malpighian tubules were dissected in PBS containing 5 mg/ml proteinase K to gently remove epithelium and to expose mineral deposits inside the tubule lumen as previously described^18^, air-dried on carbon support, coated with 5 nm chromium, and imaged using a Hitachi SU70 scanning electron microscope, followed by energy-dispersive X-ray-spectroscopy (EDX) microanalysis an individual microsphere.

### Data analysis

Student’s t-test or ANOVA with Tukey’s correction for multiple comparisons was used to determine significant differences between two or more than two treatment groups with significance threshold p < 0.05, respectively.

## Supplementary information

Supplementary info

Table S2

Table S3
