# Supplementary material for: Endocrine regulation of MFS2 by branchless controls phosphate excretion and stone formation in Drosophila renal tubules
Source: Sci Rep. 2019 Jun 19;9:8798. doi: 10.1038/s41598-019-45269-x (PMC6584732; doi:10.1038/s41598-019-45269-x)
Supplement: Supplementary file 1 — Supplementary info [file 41598_2019_45269_MOESM1_ESM.docx]

**Supplemental information for “Endocrine regulation of *MFS2* by *branchless* controls phosphate excretion and stone formation in *Drosophila* renal tubules** Emily Rose, Daniela Lee, Emily Xiao, Wenzhen Zhao, Mark Wee, Jonathan Cohen, Clemens Bergwitz”

**Table S1: Primers**

| ID | 5’ to 3’ sequence | orientation | Target (gene) amplicon length |
| --- | --- | --- | --- |
| 462 | ACAGATACAGGCGGATCGAC | F | *MFS2 (FBgn0016684)* *358 bp* |
| 463 | CACGATGAAACCGATGAATG | R | *MFS2 (FBgn0016684) 358 bp* |
| 464 | CGTGAGTGGAATGATTGGTG | F | *MFS2 (FBgn0016684) 170 bp* |
| 465 | GCAATTTGGATTCCGAGAAA | R | *MFS2 (FBgn0016684) 170 bp* |
| 711 | TCCTCAAGCAGAATCGACCT | F | *btl (FBgn0005592) 231 bp* |
| 712 | AATCGGCGATCTTCATAACG | R | *btl (FBgn0005592) 231 bp* |
| 713 | CGAAGAATACAACGCGTCCA | F | *bnl (FBgn0014135) 143 bp* |
| 714 | TTAGGGAGCGAGGAATGAAG | R | *bnl (FBgn0014135) 143 bp* |
| 727 | GCGAGCTACGAGGACTCAAG | F | *bnl (FBgn0014135) 233 bp* |
| 728 | CGAGACTCCGTCGTAATACATTG | R | *bnl (FBgn0014135) 233 bp* |
| 697 | ATGCTAAGCTGTCGCACAAATG | F | *Rpl32 (FBgn0002626) 107bp* |
| 698 | GTTCGATCCGTAACCGATGT | R | *Rpl32 (FBgn0002626) 107bp* |

**Table S2: Survey of tissue expression of candidate PFA-sensitive transporters**

Using FlyAtlas ^1^(<http://flyatlas.org/>) absolute and relative mRNA expression of various transporters previously reported by us (see Tables S3 in ^2^) is shown for various larval and adult fly tissues.

**Table S3: *Drosophila* orthologs for human regulators of Pi homeostasis**

Using the gene list indicated in tab 1 the DRSC Integrative Ortholog Prediction Tool (DIOPT v. 7.1 (March 2018)) was searched on 10/31/2018 and results are shown in tab 2. For documentation see http://www.flyrnai.org/diopt

**Figure S1:** **A:** Wild type *yellow white (y w)* flies were cultured on control medium, or medium containing the Pi transport inhibitor phosphonoformic acid 5mM (PFA) at 29°C for 30 days followed by dissection**.** PFA does not affect tubule mineral deposits despite raising hemolymph Pi (see Fig. 1B). **B:** Using FlyAtlas ^1^(<http://flyatlas.org/>) mRNA expression of *MFS2* (encoded by *FBgn0016684*) is shown for various larval and adult fly tissues.

**Figure S2: Tubule micrographs**

**A:** Flies expressing the principle cell driver *Uro-Gal4^ts^* or stellate cell driver *Gal4^c724,ts^* and RNAi targeting *MFS2* or *white (w)* as control or a transgene (TG) for *bnl* (*bnl^TG^*) were generated as shown in in the scheme, cultured on control medium or medium containing 30 mM sodium phosphate (P30) at 29°C to induce gene expression for 30 days followed by dissection**.** Ablation of *MFS2* in stellate cells has no effect (**B**), while ablation of *MFS2* in principal cells blocks formation of tubule stones (**C**). Overexpression of *bnl* stimulates tubule stone formation (**D**). Representative micrographs of 4-8 tubule pairs (arrow points to collecting duct, * marks calcium Pi deposits in distal tip of tubules).

**Figure S3: Amino acid alignment of human FGF23 with *bnl, pyr, ths***

**Legend: A:** using FGF23 as search term in the DIOPT online tool (accessed 3/17/19) a 118 amino acid core region corresponding to position 53 to 165 of human FGF23 showed 33% identity and 49% similarity. **B** Using CLUSTAL O (1.2.4) a multiple sequence alignment for human FGF23_Q9GZV9 and bnl_Q86NQ2 found 23.27% identity, between human FGF23_Q9GZV9 and ths_Q6Q7I9 found 13.33% identity, and between human FGF23_Q9GZV9 and pyr_B9ZW35 found 13.08% identity.

**Definitions for Figure S3B:**

**Alignment Conservation Annotation** This is an automatically calculated quantitative alignment annotation which measures the number of conserved physico-chemical properties conserved for each column of the alignment. Its calculation is based on the one used in the AMAS method of multiple sequence alignment analysis : Livingstone C.D. and Barton G.J. (1993), Protein Sequence Alignments: A Strategy for the Hierarchical Analysis of Residue Conservation. CABIOS Vol. **9** No. 6 (745-756)). [*View an HTML version of the paper*](http://www.compbio.dundee.ac.uk/papers/amas/amas3d.html)

Conservation is measured as a numerical index reflecting the conservation of [physico-chemical properties](about:blank../misc/aaproperties.html) in the alignment: Identities score highest, and the next most conserved group contain substitutions to amino acids lying in the same physico-chemical class.

Conservation is visualised on the alignment or a sequence group as a histogram giving the score for each column. Conserved columns are indicated by '*' (score of 11 with default amino acid property grouping), and columns with mutations where all properties are conserved are marked with a '+' (score of 10, indicating all properties are conserved).

Mousing over a conservation histogram reveals a tooltip which contains a series of symbols corresponding to the physicochemical properties that are conserved amongst the amino acids observed at each position. In these tooltips, the presence of ! implies that the lack of a particular physicochemical property is conserved (e.g. !proline).

**Alignment Quality Annotation:** Alignment Quality is one of the automatically calculated quantitative alignment annotations displayed below the columns of a multiple sequence alignment (and can be used to shade the alignment). It is an ad-hoc measure of the likelihood of observing the mutations (if any) in a particular column of the alignment. More precisely, the quality score is inversely proportional to the average cost of all pairs of mutations observed in a particular column of the alignment - a high alignment quality score for a column would suggest that there are no mutations, or most mutations observed are favourable.

The Algorithm The quality score is calculated for each column in an alignment by summing, for all mutations, the ratio of the two BLOSUM 62 scores for a mutation pair and each residue's conserved BLOSUM62 score (which is higher). This value is normalised for each column, and then plotted on a scale from 0 to 1. Multiple alignment algorithms using the BLOSUM 62 substitution matrices should, in theory, maximise alignment quality for an un-gapped alignment, and locally maximise quality for gapped alignments.

**Alignment Consensus Annotation**: The consensus displayed below the alignment is the percentage of the modal residue per column. By default this calculation includes gaps in columns. You can choose to ignore gaps in the calculation by right clicking on the label "Consensus" to the left of the consensus bar chart. If the modal value is shared by more than 1 residue, a "+" symbol is used in the display for the simple reason that it is not possible to display multiple characters in a single character space.

**Alignment occupancy:** Occupancy annotation row shows number of ungapped positions in each column of the alignment

1 Chintapalli, V. R., Wang, J. & Dow, J. A. Using FlyAtlas to identify better Drosophila melanogaster models of human disease. Nat Genet 39, 715-720, htpps://doi.org/10.1038/ng2049 (2007).

2 Bergwitz, C. *et al.* Roles of major facilitator superfamily transporters in phosphate response in Drosophila. PLoS One 7, e31730, htpps://doi.org/10.1371/journal.pone.0031730 (2012).
